# Success Factors of Medical Crowdfunding Campaigns: Systematic Review

**DOI:** 10.2196/30189

**Published:** 2022-03-22

**Authors:** Xiaoyan Hou, Tailai Wu, Zhuo Chen, Liqin Zhou

**Affiliations:** 1 School of Medicine and Health Management Huazhong University of Science and Technology Wuhan China; 2 School of Economics Faculty of Humanities and Social Sciences University of Nottingham Ningbo Ningbo China; 3 Department of Health Policy and Management College of Public Health University of Georgia Athens, GA United States

**Keywords:** medical crowdfunding, success factor, systematic review, methodology, theories, preconceptions, crowdfunding, fundraising, financial resources, health care

## Abstract

**Background:**

Medical crowdfunding provides opportunities for individuals who lack financial resources to access the health services that they need. Despite the popularity of medical crowdfunding, the current understanding of the success of medical crowdfunding campaigns is fragmented and inadequate.

**Objective:**

We aimed to comprehensively investigate which factors lead to the success of medical crowdfunding campaigns.

**Methods:**

A search was conducted in PubMed, PsycINFO, Web of Science, ACM Digital Library, and ScienceDirect from 2010 to June 2020. Papers directly and indirectly related to the success of medical crowdfunding campaigns were included. Two reviewers independently extracted information on the success of medical crowdfunding campaigns.

**Results:**

Our search yielded 441 articles, of which 13 met the inclusion criteria. Medical crowdfunding is increasingly attracting academic attention, and most studies leverage text analysis as their research methods; however, there is a lack of consensus on the definition of medical crowdfunding among researchers. Four categories of factors that affect the success of medical crowdfunding were identified: platforms, raisers, donors, and campaigns.

**Conclusions:**

Although some limitations exist in our systematic review, our study captured and mapped literatures of the success of medical crowdfunding campaigns systematically, which can be used as the basis for future research on this topic.

## Introduction

Crowdfunding has a massive impact on how people access health care services [1]. Due to the low requirements and easy set-up of crowdfunding websites, the usage of crowdfunding platforms for health-related fundraising is growing increasingly popular. Crowdfunding platforms, such as GoFundMe, Kickstarter, and FundRazr, are highly utilized for raising funds for a variety of causes, especially medical needs [2]. Some crowdfunding websites report that the category *medical campaigns* ranks as the top-grossing [3]. The usage of crowdfunding websites for medical expenses is expected to increase by 25% annually [3]. Web-based medical crowdfunding emerged after 2008, during which the economic crunch led to an equally enthusiastic increase in the use and accessibility of social media platforms [4]. Medical crowdfunding is donation-based and is used to raise money for those who have medical costs. Medical crowdfunding may represent how people respond to the gaps in national health payment systems [5]; many health needs that are not met by national health insurance coverage are reflected in medical crowdfunding platforms [5-7]. The lower the national insurance coverage, the greater the number of medical crowdfunding projects [8]. The benefits of medical crowdfunding include expanding funder participation in the health market, improving the access to financial support, drawing funding to neglected health issues, and improving social engagement [9]. Medical crowdfunding also has been shown to reduce the rate of personal bankruptcy [10]; therefore, it is becoming an important way to deal with medical financial issues.

Generally, the success of a medical crowdfunding campaign could be defined as the degree to which the medical crowdfunding campaign achieves or exceeds the goals set by fundraisers. However, despite the convenience and popularity of medical crowdfunding websites, the success rates of medical crowdfunding campaigns on different platforms vary dramatically. For example, in China, campaigns have been reported to achieve just 18% of their goals [11], whereas, in the United States, medical crowdfunding campaigns have achieved over 40% of their goals on average [4]. Overall, only 10% of medical crowdfunding campaigns have been reported to reach their fundraising target [4], and some campaigns reach their fundraising targets within a short time, while others struggle to raise their target amount. Success factors of medical crowdfunding campaigns are factors that lead the campaigns to achieve or exceed the target amount [12]. Thus, knowing these factors could help improve the success rate of medical crowdfunding campaign.

Medical crowdfunding can not only make up for the deficiencies of health insurance systems but can also address problems such as limited financing channels and low private capital utilization rates [1,13,14]. However, the negative consequences of medical crowdfunding have also become apparent and have been increasing [3,15-17]. Factors such as low-entry barriers to launch or donate to campaigns, too much separation between raisers and donors, and anonymity could increase the risk of fraud in medical crowdfunding campaigns [18,19]. In addition, the current understanding of medical crowdfunding success factors is limited. The importance and potential issues of medical crowdfunding have stimulated the interest of academic researchers [20]. Although the success factors of medical crowdfunding campaigns have been previously considered, each study has examined the factors from different perspectives and using samples from different countries. For example, Durand et al [12] analyzed success factors only from text features. Thus, current literature on the success factors of medical crowdfunding campaigns is segmented, lacking the ability to give a holistic understanding of the factors. A systematic review [21] was needed to systematically search, critically appraise, and synthesize studies, to explore the success factors of medical crowdfunding campaigns. We aimed to comprehensively and systematically investigate the factors leading to the successes of medical crowdfunding campaigns.

## Methods

### Overview

We chose to conduct a systematic review, rather than choosing another method, for the following reasons: First, a systematic review could provide a comprehensive understanding concerning the success factors of medical crowdfunding campaigns. The comprehensive understanding could be a solid basis for further studies on this topic. Second, a systematic review could limit bias in identifying and rejecting bias by using explicit methods. Third, conclusions of the systematic review may be more reliable and accurate because it summarizes previous literature systematically. Fourth, the findings of systematic review could be used to reduce the delay between scientific discoveries and implementation. The generalizability of findings could be established by comparing studied success factors of medical crowdfunding campaigns [22].

### Literature Search

We used PubMed, PsycINFO, Web of Science, ACM Digital Library, and ScienceDirect databases. These databases were chosen because they cover most disciplines that study medical crowdfunding, namely, medicine, information, psychology, global health, computer science, and business economics. Keywords and synonyms used in this search revolved around 2 concepts—crowdfunding, and health (Textbox 1). Database searches were conducted on June 8, 2020 by 2 authors (LZ and XH) with Library and Information Science expertise. The results were exported to Zotero (version 5.0.0.0; Corporation for Digital Scholarship) and organized into folders by database. PRISMA (Preferred Reporting Items for Systematic Reviews and Meta-analyses) was used to document the review process was used.

**Search strategy. The terms were searched in keyword, Boolean/phrase search modes, and all fields, within PubMed, PsycINFO, Web of Science, ACM Digital Library, and ScienceDirect respectively**

- 11 crowdfunding or crowd funding
- 22 health or disease or illness or medical or hospital or treatment
- 33 1 and 2

### Inclusion and Exclusion Criteria

Papers that focused on the success of medical crowdfunding or other factors related to the success of crowdfunding (eg, narrative strategies and ethical factors of medical crowdfunding) published between 2010 and 2020 (because medical crowdfunding emerged after 2008) within conference proceedings or journals were included, and papers without research design details or results or not be written in English were excluded.

### Quality Assessment

Papers were rated for quality using the McGill Mixed Methods Appraisal Tool version 2018 [23]. Its internal reliability, usability, and content validity have been verified in several studies [24,25]. Quality criteria are applied based on the study design (qualitative research, quantitative research, mixed method research) and methodology (such as randomized controlled, nonrandomized and descriptive studies). We used criteria [23] for quantitative descriptive studies: (1) Is the sampling strategy relevant to address the quantitative research question? (2) Is the sample representative of the population understudy? (3) Are measurements appropriate? (4) Is there an acceptable response rate? We also used a quality threshold (75%, ie, meeting at least 3 criteria) defined by a previous study [26]. Papers that did not meet the threshold were excluded.

### Data Analysis

Metadata were extracted and listed in a spreadsheet (Excel, Microsoft Inc): author region, publication date, data sources, research questions, data collection settings, methods, results, discussions, conclusions, and bibliographies. To interpret our findings, all authors discussed how to present our findings systematically. Since the conception of medical crowdfunding is dynamic, we discuss the conception, then summarize bibliographic and study information. The success factors are based to the contextual structure of medical crowdfunding. Since there are 3 main actors in medical crowdfunding, and they interact with each other based on specific campaigns [7], we categorized the success factors by platforms, raisers, donors, and campaigns. Because of the heterogeneity of outcome measures in studies included in our review, it was impractical to conduct a meta-analysis. Therefore, findings were qualitative.

## Results

### Search Results

**Figure 1:**
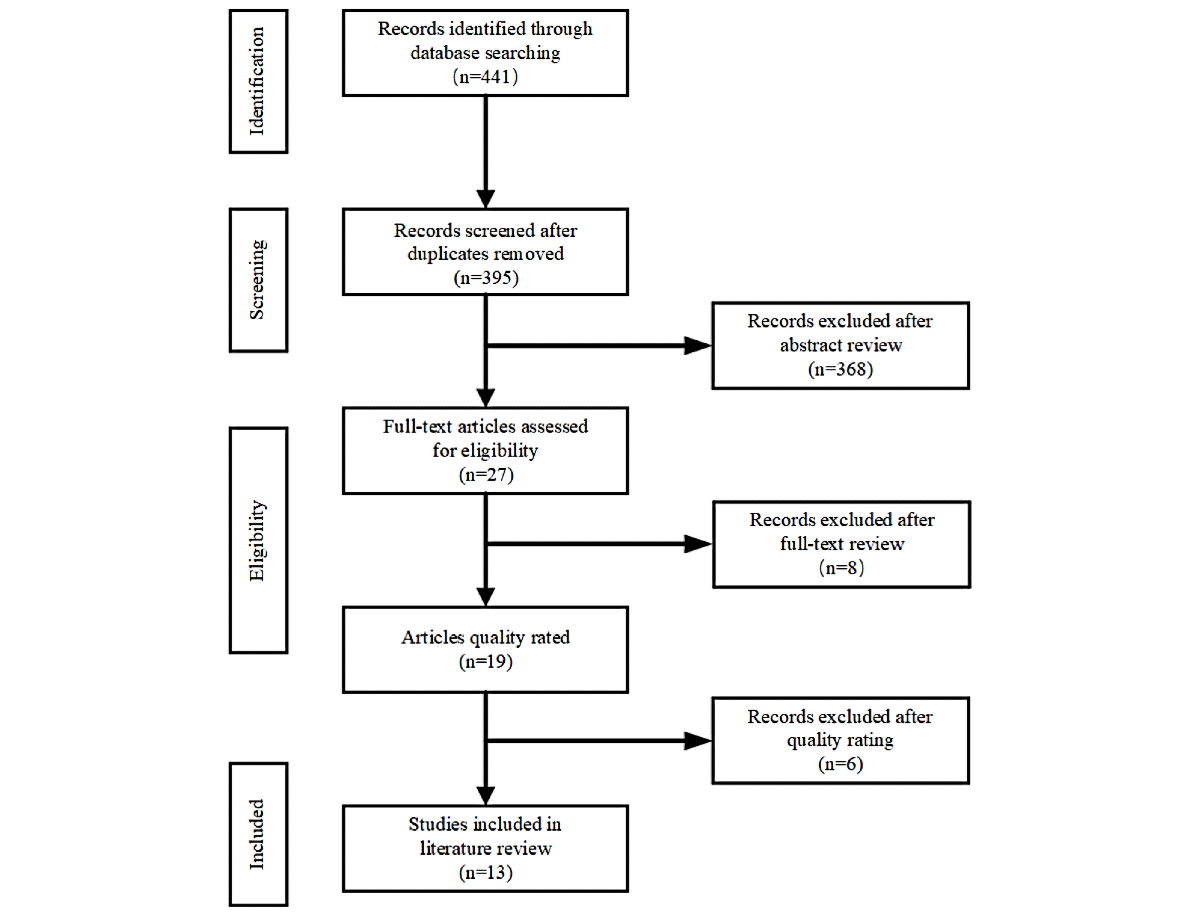
Summary of literature search and study selection process.

A total of 441 papers were identified from the 5 databases. After removing 46 duplicates, titles and abstracts were screened by 2 authors separately. When a difference in opinion arose, a third author was involved to mediate the discussion to reach agreement. Using inclusion and exclusion criteria, 27 papers were chosen for full-text review. After full-text review, 19 papers were assessed for quality, 13 papers were included (Figure 1).

### Medical Crowdfunding Preconceptions

Despite the acknowledgment of the benefit of medical crowdfunding in strengthening health care, there is still a lack of consensus on what constitutes crowdfunding campaigns in health care. However, to study the success factors of medical crowdfunding, we need to have a basic understanding of medical crowdfunding. Based on previous studies [6-8], we define the scope of medical crowdfunding as crowdfunding for medical expenses in hospitals, new drugs in scientific research institutions, and new treatments (eg, hospitalization expenses, scientific research funds).

### Description of the Studies

Of the 13 studies (Table 1), 9 studies [4,8,12,27-32] relied entirely on crowdfunding websites such as GoFundMe. There were 4 main analytical methods used: text analysis, regression analysis, semistructured interviews, and exploratory spatial analysis. Text analysis was used most, with 4 studies using only text analysis [4,29-31], and 2 studies using text analysis partially [12,33]. Of the 13 studies, 6 studies were directly related to the success of medical crowdfunding, and the remaining 7 studies were indirectly related. The number of publications appears to be steadily increasing over time, which indicates that this field is getting more and more attention from scholars and practitioners because of the particularity of medical crowdfunding [6,20]. Most studies were from high-income countries, with the largest source of articles being the United States [4,12,27,32,33], followed by Canada [30,34].

**Table 1:** Study information.

Reference	Publication date (year, month)	Country	Study aim	Data sources	Method	Success factors
Durand et al [12]	2018,6	United States	To identify the factors influencing the success of crowdfunding campaigns	YouCaring	Text analysis, multiple linear analysis, logistic regression analysis	Campaign description length; goal amount; third-person description perspective; cognitive state
Kim et al [27]	2018,4	United States	To investigate how beneficiaries present their situations and how contributors view the information presented	GoFundMe, YouCaring, Fundly	Semistructured interviews	Authenticity
van Duynhoven et al [34]	2019,6	Canada	To explore the role of socioeconomic status in medical crowdfunding campaigns	Cancer-related activities published by Canadians; The 2016 Census Profile for aggregate dissemination area and area boundaries; forward sortation area boundaries	Exploratory spatial analysis	Socioeconomic status; demographic
Xu and Wang [28]	2019,8	China	Make clear the narrative strategy of medical crowdfunding article	Easy Fundraising	Thematic narrative analysis	Narrative strategies
Kim et al [33]	2016,5	United States	To assess the credibility of web-based medical crowdfunding campaigns	(1) Comment on Reddit related to the medical crowdfunding campaign, (2) 20 participants	Text analysis and semistructured interviews	Credibility; individual prestige
Aleksina et al [35]	2019,7	France	To investigate the determinants of successful crowdfunding campaigns in medical research	Consano, Experiment	Ordinary least square regression	Number of tweeters; goal amount; platform availability; total campaign number; total fundraising amount; total donor number
Holmes et al [29]	2019,4	United Kingdom	To determine whether crowdfunding of pharmacy-related products through popular web-based platforms	Kickstarter, Indiegogo	Text analysis	Media attention; platform audit; demographic information of donors
Snyder et al [30]	2017,6	Canada	To explore how Canadians can demonstrate to others that they should fund their health needs	FundRazr, Generosity, GoFundMe, YouCaring	Text analysis	Personal connections; depth of need; giving back; ethics
Koole et al [31]	2018,12	Netherlands	Identify key factors for the success of crowdfunding for grown-up congenital heart patients	A web-based donation platform	Text analysis	Professional organization support; stakeholder support; easy-to-understand message
Barcelos and Budge [32]	2019,1	United States	Investigated how transgender communities utilize crowdfunding expenses related to gender afﬁrming medical care	GoFundMe	Hierarchical multiple regression analyses	Social media (Facebook) sharing; demographic information of raiser (age, location, race, identity)
Berliner et al [4]	2017,8	United States	Explore the usage, impacts, or consequences of the increasing reliance on crowdfunding for health	GoFundMe	Text analysis	Medical literacy; media literacy
Bassani et al [8]	2019,8	Italy	Examine the worldwide population of health care crowdfunding platforms and explore the relationship between health care crowdfunding success and national health systems	76 crowdfunding platforms that host health care campaigns	Negative binomial regressions	Platform type; social return
Kenworthy [36]	2019,11	Norway	Map and document how medical crowdfunding is shaped by, and shapes, health disparities	An ethnography of US medical crowd-funding; a study of global health crowdfunding; a project of US medical crowdfunding campaigns	Exploratory conceptual and empirical analysis	Platform design (Search engine, lists, webpage, etc); Partnership with traditional media; Deservingness; Narratives

### Success Factors

#### Platforms

For platforms, the factors influencing the success of medical crowdfunding campaigns can be divided into 2 aspects: technical and social (Table 2).

The *technical aspect* reflects the functionality of medical crowdfunding platform in terms of platform audit, platform availability, platform types and platform design. *Platform audit* is the review of campaigns by platforms and could impact donor’s decision on donation. Review of campaigns by crowdfunding platforms is closely related to the eventual success of the crowdfunding campaigns [2]. *Platform availability* is the degree to which a medical crowdfunding platform is available to their users. The availability of the platform positively affects donor incentive, and thus, project funding results [36]. In addition, the type of platform can also have impact on fundraising. Platforms with more extensive publicity are more popular than specialized, smaller platforms [8]. Platform design affects donors’ experiences—supportive behavior and word of mouth helps fundraisers attract the attention of potential donors [36].

**Table 2:** Factors influencing the success of medical crowdfunding campaigns from the platforms.

Dimensions and factors			Definitions		Functions		Reference
**Technical**							
	Platform audit	Review of campaigns on crowdfunding platforms		Reviews of campaigns by crowdfunding platforms affect donor decisions.		Holmes et al [29]	
	Platform availability	The degree to which the platform is available to users		The availability of the platform affects donor incentives, and thus, campaign funding results.		Aleksina et al [35]	
	Platform types	Whether the platform is specialized or general		Platforms with more extensive publicity are more popular than specialized smaller platforms.		Bassani et al [8] Aleksina et al [35]	
	Platform design	Design elements of the platform including search engine, lists, webpage, etc		Platform design determines donors’ experiences, and consequently, their donation behavior.		Kenworthy [36]	
**Social**							
	Total campaign number	Total number of campaigns initiated in the platform		With more categories and projects, more potential donors visit the platform.		Aleksina et al [35]	
	Total fundraising amount	Total money raised in the platform		Higher total amounts raised on the platform represent higher recognition and acceptance of the platform.		Aleksina et al [35]	
	Total donor number	Total number of donors appears in the platform		With more donors in the platform, there is a better possibility of getting funding.		Aleksina et al [35] Barcelos and Budge [32]	
	Partnership with traditional media	Platform collaboration with traditional media to disseminate the information of its campaigns		Traditional media can help medical crowdfunding campaigns get more donors.		Kenworthy [36]	

The *social aspect* reflects interactions inside and outside the platform in terms of total campaign number, total fundraising amount, total donor number and total and partnership with traditional media. The larger the number of categories and campaigns contained, the larger the number of potential donors attracted by the platform, and the greater the probability of receiving donations [37]. If the total amount of fundraising on the platform is higher, fundraisers who publish campaigns on the platform have more confidence that they will raise the amount they want [35]. Moreover, to some extent, the total number of donors can represent the amount that could be raised [38]. The number of registered institutions in the platform also reflects the platform’s position within the industry and the resources that are available to campaigns in the platform [3]. In addition, partnerships with traditional media allow campaigns to have opportunities to access mass media and their audiences, increasing the number of potential donors. With more interactions, there is more social capital, with higher fundraising possibilities [39]. The larger the potential donor base, the higher the possibility of reaching the target amount [9].

#### Raisers

The influencing factors from raisers (ie, the beneficiaries) can be analyzed in terms of 3 aspects: demographic, individual characteristics, and social (Table 3).

The demographic characteristics of the raisers include age, nationality, and geographic locations. Younger individuals were more likely to succeed in health crowdfunding [32]. Location was also an important factor—raisers in remote areas are less successful in raising money, while raisers in affluent areas, in there are more social resources, are more likely to succeed in raising money [37]. Moreover, raisers with higher levels of education and higher income were shown to be more likely to attract the attention of potential donors and thus get donations [34].

Successful crowdfunding campaigners had better media literacy and medical literacy [4]. Raisers with high media literacy are more likely to spread their message across social media platforms and attract potential donors. Raisers with higher medical literacy are more likely to provide accurate disease-related and health care information [4]. In addition, once raisers are perceived to be deserving, their campaigns attract many donors and succeed in raising funds [36].

Social factors included personal connections, stakeholder support, professional organization support, and individual prestige. Raisers’ personal connections establish social networks and can share links, which allows more potential donors to see the information of the campaigns, thus obtaining more funds. Gaining the support of stakeholders and professional organizations makes it easier to successfully raise funds for medical crowdfunding campaigns [31], and if raisers have high individual prestige, they are more likely to gain trust and support from donors in social networks [30].

**Table 3:** Factors influencing the success of medical crowdfunding campaigns from the raisers.

Dimensions and factors			Definitions		Functions		Sources
**Demographic**							
	Age	Age		Younger people are more likely to succeed in health crowdfunding.		Aleksina et al [35]; Barcelos and Budge [32]	
	Education level	Level of education		Raisers with higher levels of education are more likely to attract the attention of donors (higher donation possibility).		van Duynhoven et al [34]	
	Income	Income		Raisers with higher income are more likely to attract the attention of donors (higher donation possibility).		van Duynhoven et al [34]	
	Geographical location	Where raisers reside		Raisers are more likely to get help from people in or near their districts, especially in wealthier places.		Barcelos and Budge [32]	
**Individual characteristics**							
	Media literacy	Raisers’ ability to make use of different media		The raisers of successful crowdfunding campaigns have good media literacy.		Berliner and Kenworthy [4]; Holmes et al [29]	
	Medical literacy	Raisers’ ability of leverage different medical knowledge		A certain level of medical literacy (of raisers) facilitates the proper description of the disease and relevant understanding of the health care system.		Berliner et al [4]	
	Deservingness	The degree to which raisers are thought to be deserving of receiving donations		Once raisers are perceived to be deserving, their campaigns attract many donors, and they succeed in raising funds.		Kenworthy [36]	
**Social**							
	Personal connections	Raisers’ personal connections with others including their families, friends, and colleagues		The scale of raisers’ personal connections has a positive effect on the success rate of fundraising.		Snyder et al [30]	
	Stakeholder and professional organization support	Raisers get support from different stakeholders and professional organizations		The support from stakeholders and professional organizations makes fundraising easier.		Koole et al [31]	
	Individual prestige	Raisers’ personal respect and admiration from others inside and outside the platform		The prestige of raisers can serve as the signal of the credibility and success of their campaigns.		Kim et al [33]; Snyder et al [30]	

### Donors

The determinants of intention to donate in donors can be mainly divided into demographic and individual characteristics (Table 4).

Donor gender [40,41], age [29], education level [29], income [29], and geographical location [35] had significant effects on donation behaviors. Younger donors were more willingness to donate [32], and people with higher education and income level were more likely to donate [42]. Donors were more willing to contribute to crowdfunding campaigns in or near their own regions [35]; therefore, geographic inequity exists, which is compounded by social, technological, and cultural issues [4].

Individual characteristics included cognitive state and social returns. *Cognitive state* is state invoked in the donor in reading a campaign’s description [12]. If donors feel threatened by reading the negative campaign descriptions, they hesitate to donate. When donors are aware the importance and urgency of the raiser's medical crowdfunding campaigns, they have higher willingness to donate [43]. *Social return* is a major intrinsic motivation for individuals or groups to donate to medical crowdfunding campaigns [4]. People with prosocial values and who participate more in charitable activities donate more willingly [44]. Donors with intrinsic motivation have high willingness to donate.

**Table 4:** Factors influencing the success of medical crowdfunding campaigns from donors.

Dimensions and factors			Definitions		Functions		Sources
**Demographic**							
	Age	Age		Younger donors have higher willingness to donate.		Holmes et al [29]	
	Education level	Level of education		Donors with higher levels of education have higher willingness to donate.		Holmes et al [29]	
	Income	Income		Donors with higher income have higher willingness to donate.		Holmes et al [29]	
	Geographical location	Where raisers reside		Donors are more willing to contribute to crowdfunding campaigns in or near their own regions.		Aleksina et al [35]; Berliner and Kenworthy [4]	
**Individual characteristics**							
	Cognitive state	The cognitive state of the donor when they read the campaign		Donors may feel threatened by negative campaign descriptions and social pressure to donate. The positive cognitive state invoked by the campaign description would promote donors’ donation behavior.		Durand et al [12]	
	Social returns	The intrinsic motivation of donors to give back to society		The more returns to the society from the donation, the more possibility donors would donate.		Bassani et al [8]	

### Campaigns

Success factors for campaigns can be divided into 2 aspects: the format and the content (Table 5).

Format-related factors include goal amount, campaign description length, third-person description, and social media sharing. When a campaign is close to its fundraising goal, it encourages donation behavior and increases the likelihood of project success [40,45]. Longer campaign descriptions and higher goal amount were significantly associated with amount raised [12]. In addition, third-person perspective in the description can convey a patient’s positive qualities in a way that would be not acceptable in the first-person perspective due to the testimonial effect [12]. It is also critical for crowdfunding campaigns to leverage social media [35]. The amount raised was strongly correlated with updates and shares in social media [12]. One additional tweet or retweet with more personal comments could enhance the probability of success of crowdfunding campaigns [35].

Content-related factors included the narrative strategy, authenticity, credibility, being easy to understand, giving back, and depth of need. *Illness narratives* represent the personal story and illness experience that patients are sharing verbally or in writing [28]. The narration style not only affected the efficiency of the dissemination of health information but also heavily affected on the potential donors' cognitions, attitudes, and behaviors [28]. Authenticity, which can be conveyed by pictures of raisers that depict their medical conditions, increases the possibility of donations. Funding is more accessible when the presented narratives of campaigns were credible [46]. To demonstrate the credibility of campaigns, many methods, including collective endorsements, presenting details of external financial support, displaying off-site verification details (of ailment, incident, and treatment), providing verification of fundraiser and beneficiary identities, and using a popular and trusted platform, can be used [33].

Easy-to-understand information in the campaign description gives potential donors a clear picture of the campaign [31]. In addition, portraying the beneficiary as someone who selflessly gives back to society not only helps establish the positive image of raisers, but also, inspires the donors themselves [30]. Patients in urgent need of funds because of disease or for treatment are easy to obtain donations from potential donors [30].

**Table 5:** Factors influencing the success of medical crowdfunding campaigns from campaigns.

Dimensions and factors		Definitions	Functions	Sources
**Format**				
	Goal amount	The objective amount of money the campaign plan to raise.	Goal amount has a positive impact on campaign success.	Durand et al [12]; Aleksina et al [35]
	Campaign description length	The length of the medical crowdfunding campaign description.	Campaign description length has a positive impact on campaign success.	Durand et al [12]
	Third-person description perspective	The narrative perspective of the campaign is the third person.	The third-person perspective makes the story more objective and realistic, which makes it more convincing.	Durand et al [12]
	Social media sharing	The number of shares and likes of campaigns in social media which connect to the platform.	The more shares and likes potential donors see, the more likely they are to donate.	Barcelos and Budge [32]
**Content**				
	Narrative strategy	The way that raisers describe their illness and ask for donations.	Narrative strategies such as more positive emotions, more information, and appropriate arousal level have impacts on crowdfunding success.	Durand et al [12]; Koole et al [31]; Kenworthy [36]
	Authenticity	The content of medical crowdfunding campaigns is authentic.	Potential donors payed attention on their impression of raisers’ authentic medical conditions and make their decisions based on it.	Kim et al [27]
	Credibility	Credibility of medical crowdfunding campaigns.	Credibility of campaigns which could be formed based collective endorsement have impacts on the success of medical crowdfunding campaigns.	van Duynhoven et al [34]; Kim et al [33]; Koole et al [31]
	Easy-to understand message	The degree to which the message is easily understood.	Easy-to-understand information helps potential donors get a sense of the raisers’ intention, which in turn helps donors make decisions.	Koole et al [31]
	Giving back	Portraying the raisers as someone who selflessly gives back to society.	The past efforts of the raisers on behalf of others were used as a rationale for the potential donors to contribute to the crowdfunding campaign.	Snyder et al [30]
	Deep of need	Campaign content reflects the urgent need of funds to solve health problems.	The urgency of need for help would determine the success of raising money.	Snyder et al [30]

## Discussion

### Principal Findings

By investigating 13 studies, the key factors of successful medical crowdfunding campaigns were extracted. On this basis, we conducted a more in-depth review and provide a comprehensive understanding of the factors that influence successful campaigns. We find the success factors of medical crowdfunding campaigns could be divided into 4 categories: platforms, raisers, donors, and campaigns. The success factors involve the main actors in the whole medical crowdfunding campaigns, but campaign factors were more frequently studied than other factors. Platform factors, however, played an important role in medical crowdfunding [47], because platforms are important in linking donors and raisers [48]. There were relatively few studies related to donors; thus, future studies could pay more attention to this area. Despite the widespread use of medical crowdfunding, to the best of our knowledge, this is the first systematic review that examines the factors that lead to successful medical crowdfunding campaigns.

### Implications

The 4 categories of successful factors that are actors in medical crowdfunding interact with each other [7,49,50]. First, raisers register in the platforms to initiate campaigns to raise money for medical expenses with illness narratives, which include fundraising goals, fundraising time, and fundraising events. Second, the medical crowdfunding platforms conduct a preliminary review to release the campaigns. Third, managers of the platforms give the funds raised from the public to the campaigners after the relevant processing charges are collected. A crowdfunding campaign will be success if all the above steps are were successfully implemented.

Theory for understanding the success factors of medical crowdfunding can be developed from our findings. None of articles included in this review take strong theoretical perspective to help analyze, explain, and predict the success factors of medical crowdfunding and convey few theoretical implications. Therefore, it is necessary to apply more theories to better understand this topic. Second, more methods or mixed methods could be employed to investigate this topic. Although text analysis was used most often, more insights could be explored using other methods that possess different advantages. We found that data were collected from single sources, which may result inherent biases; collecting data from multiple sources could allow validation to proposed research models and used methods in different studies.

### Limitations and Future Directions

Our study has some limitations. First, no new factors and relationships about medical crowdfunding could be explored through systematic review. This was the most significant inherent limitation of our study since we relied on previously published literature. Second, additional literature could have been included in our systematic review. Literature written in other languages from other databases may also have discussed the success factors of medical crowdfunding. Third, bias may still exist in the process of selection and evaluation of literature. Although we attempted to minimize bias from differences between reviewers, such as solving conflicts in judgment by discussion among reviewers, bias cannot be excluded. Fourth, heterogeneity exists in the literature included in the review; therefore, the quality of our review results may be impacted. Other categories of success factors, such as health systems or national economic status, could be also be considered.

### Conclusion

By examining platform, raiser, donor, and campaign–related success factors, we provide information that can be used as the basis for future research and future medical crowdfunding campaigns.

## Abbreviations

PRISMA: Preferred Reporting Items for Systematic Reviews and Meta-analyses
